# Bis(4-acetoxy-*N*-ethyl-*N*-*n*-propyl­tryptammonium) fumarate–fumaric acid (1/1)

**DOI:** 10.1107/S2414314623007794

**Published:** 2023-09-08

**Authors:** Duyen N. K. Pham, Nathan B. Sackett, Andrew R. Chadeayne, James A. Golen, David R. Manke

**Affiliations:** a University of Massachusetts Dartmouth, 285 Old Westport Road, North Dartmouth, MA 02747, USA; b University of Washington, Department of Psychiatry & Behavioral Sciences, Center for Novel Therapeutics in Addiction Psychiatry, 1959 NE Pacific Street, Box 356560, Seattle, WA 98195, USA; c CaaMTech, Inc., 58 East Sunset Way, Suite 209, Issaquah, WA 98027, USA; University of Aberdeen, United Kingdom

**Keywords:** crystal structure, tryptamines, indoles, fumarates, hydrogen bonds

## Abstract

The solid-state structure of the title salt/adduct (systemic name: bis­{[2-(4-acet­yloxy-1*H*-indol-3-yl)eth­yl](eth­yl)propyl­aza­nium} but-2-enedioate–(*E*)-butenedioic acid (1/1)), 2C_17_H_25_N_2_O_2_^+^·C_4_H_2_O_4_^2−^·C_4_H_4_O_4_, was determined by single-crystal X-ray diffraction. The asymmetric unit consists of a singly protonated tryptammonium cation, one half of a fumarate dianion and one half of a fumaric acid mol­ecule. In the crystal, the ions and mol­ecules are linked together in infinite chains propagating along [001] through a series of N—H⋯O and O—H⋯O hydrogen bonds.

## Abstract 2

The structure of the synthetic psychedelic prodrug 4-hy­droxy-*N*-ethyl-*N*-*n*-propyl­tryptamine is reported as its salt with a fumarate dianion and a fumaric acid adduct.

## Graphical abstract

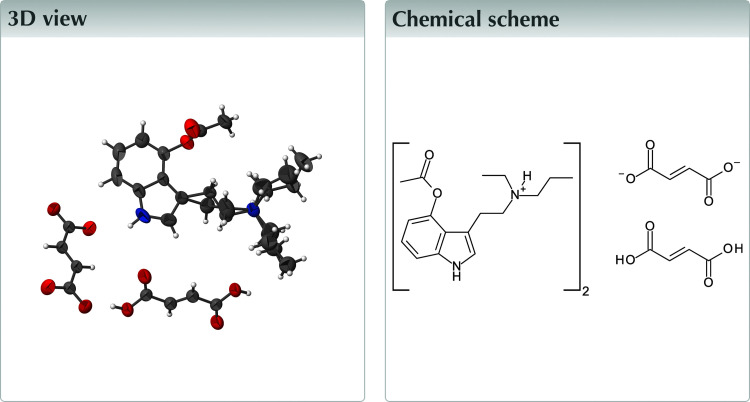

## Structure description

Psilocybin (4-phosphor­yloxy-*N*,*N*-di­methyl­tryptamine) is a natural product found in many species of mushrooms. It functions as a prodrug of psilocin (4-hy­droxy-*N*,*N*-di­methyl­tryptamine) *via* enzymatic hydrolysis of the phosphor­yloxy group to generate the active psychedelic. Psilocin is an agonist at serotonin (5-hy­droxy­tryptamine; 5-HT) receptors, most notably the serotonin 2A (5-HT_2A_) receptor, which is primarily responsible for the psychoactive and therapeutic effects of the mol­ecule. Psilocybin has shown promise in the treatment of pervasive human disorders, including depression (Carhart-Harris *et al.*, 2021 ▸; Davis *et al.*, 2021 ▸; von Rotz *et al.*, 2023 ▸), end-of-life anxiety (Grob *et al.*, 2011 ▸; Griffiths *et al.*, 2016 ▸), obsessive-compulsive disorders (Moreno *et al.*, 2006 ▸), tobacco-use disorder (Johnson *et al.*, 2014 ▸) and alcohol-use disorder (Bogenschutz *et al.*, 2022 ▸). The inter­est in psilocybin has also generated inter­est in the structure–activity relationship (SAR) of analogous compounds.

We previously reported two crystalline forms of psilacetin (4-acet­oxy-*N*,*N*-di­methyl­tryptamine) which, like psilocybin, also functions as a prodrug of psilocin. Our recent *in vivo* studies in mice demonstrate that psilacetin is more efficient than psilocybin at delivering psilocin, resulting in increased potency at equimolar amounts. This is supported by background hydrolysis rates which show psilacetin to hydrolyze forty times faster than psilocybin, but also demonstrate that the hydrolysis of either prodrug in the body must be enzymatic (Glatfelter *et al.*, 2022*b* ▸). 4-Acet­oxy-*N*-ethyl-*N*-*n*-propyl­tryptamine (4-AcO-EPT) is a putative prodrug of the synthetic psychedelic, and psilocin analogue, 4-hy­droxy-*N*-ethyl-*N*-*n*-propyl­tryptamine (4-HO-EPT). When competitive binding assays are compared, binding is observed for 4-HO-EPT across many more receptors than 4-AcO-EPT, and significantly stronger binding is observed at most receptors where 4-AcO-EPT is also competitive (Glatfelter *et al.*, 2023 ▸). 4-HO-EPT showed a substantial increase in *in vitro* functional assays for 5-HT_2A_ agonism over 4-AcO-EPT, with an observed EC_50_ of 4.24 nM, compared to an EC_50_ of 24.0 nM for the ester.

One thing that is not clear from the *in vitro* and *in vivo* studies of 4-AcO-EPT is the exact chemical composition of the experimentally studied compound. Klein *et al.* reported *in vitro* and *in vivo* data for ‘4-AcO-EPT fumarate’, which describes a doubly deprotonated di­carb­oxy­lic acid and a 2:1 molar ratio of tryptamine to fumaric acid equivalent. However, the mol­ecular weight calculations in the paper indicate that the compound studied had a 1:1 ratio of tryptamine to fumaric acid, *i.e.* 4-AcO-EPT hydro­fumarate. In our prior study, we reported 4-AcO-EPT hydro­fumarate based upon NMR data demonstrating a 1:1 ratio of tryptamine to fumaric acid equivalent, consistent with a singly deprotonated di­carb­oxy­lic acid for each tryptamine. However, this work reveals an error in this assignment, further highlighting the necessity of isolating a single crystal and performing diffraction studies when determining the exact nature of crystalline tryptamine solids. Herein we report the compound to be neither the fumarate nor the hydro­fumarate, but rather bis­(4-acet­oxy-*N*-ethyl-*N*-*n*-propyl­tryptammonium) fumarate–fumaric acid.

**Figure 1:**
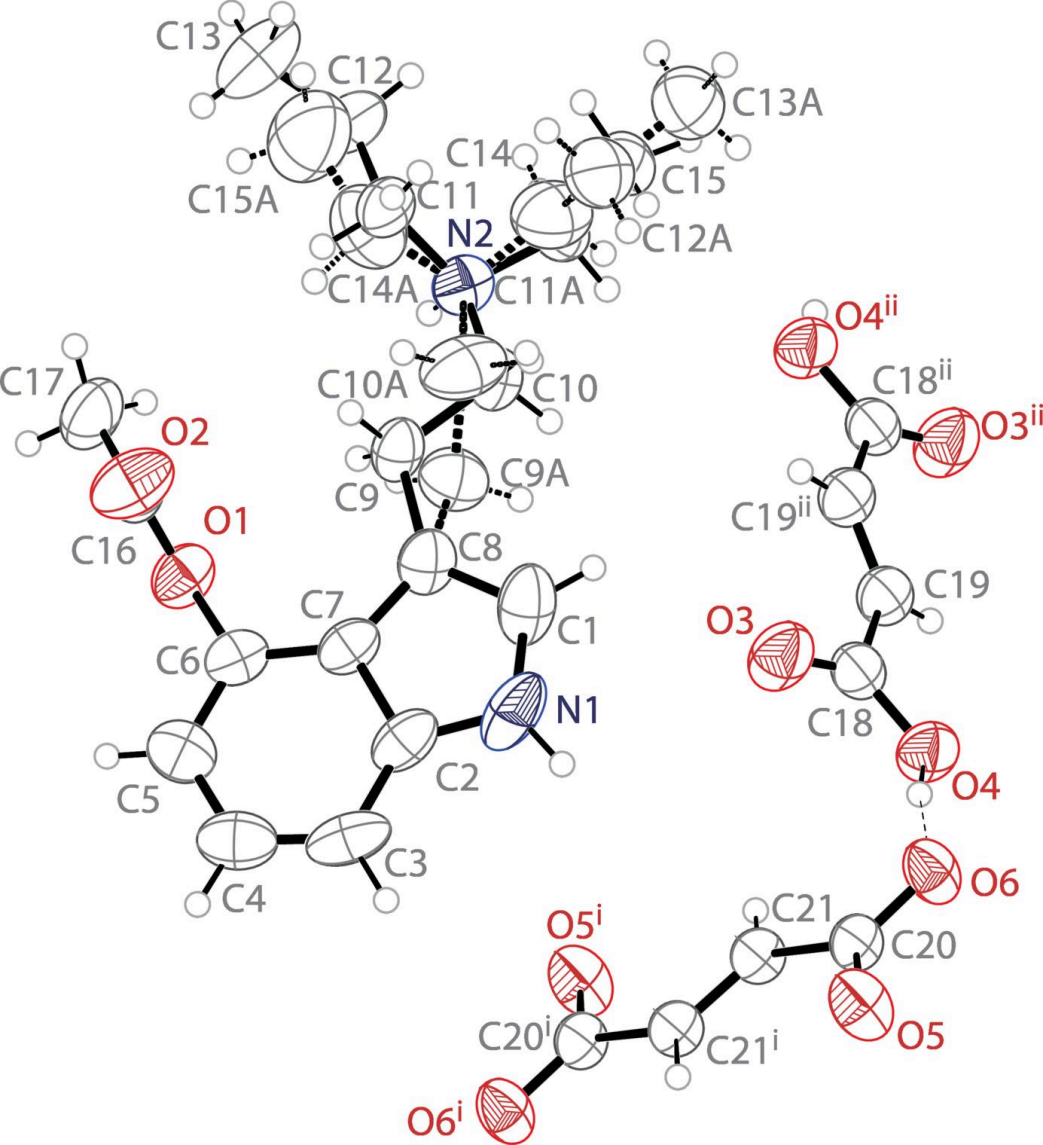
The mol­ecular structure of 4-AcO-EPT fumarate–fumaric acid showing the atomic labeling. Displacement ellipsoids are shown at the 50% probability level. Dashed bonds indicate a disordered component in the structure. Hydrogen bonds are shown as dashed lines. Symmetry codes: (i) −*x*, −*y*, 1 − *z*; (ii) −*x*, −*y*, 2 − *z*.

The mol­ecular structure of 4-AcO-EPT fumarate–fumaric acid is shown in Fig. 1 ▸. The asymmetric unit contains one 4-acet­oxy-*N*-ethyl-*N*-*n*-propyl­tryptammonium (C_17_H_25_N_2_O_2_^+^) cation, one half of a fumarate (C_2_HO_2_^−^) dianion, and one half of a fumaric acid (C_2_H_2_O_2_) mol­ecule. The indole ring system of the cation is near planar with a r.m.s. deviation from planarity of 0.018 Å. The ethyl­amino arm is disordered over two orientations in a 0.895 (7):0.105 (7) ratio. The major component of the ethyl­amino arm is nearly co-planar with the indole ring, showing a C7—C8—C9—C10 torsion angle of −176.3 (2)°. The ethyl and *n*-propyl groups are disordered over two sets of sites with a 0.741 (6):0.259 (6) ratio, with the methyl­ene carbon atoms of the *n*-propyl groups being in close proximity to the two ethyl group C atoms. The complete fumarate dianion is generated through crystallographic inversion symmetry, and is near planar with an r.m.s. deviation from planarity of 0.009 Å. The complete fumaric acid dianion in generated similarly and only slightly less planar with an r.m.s. deviation of 0.070 Å.

**Figure 2:**
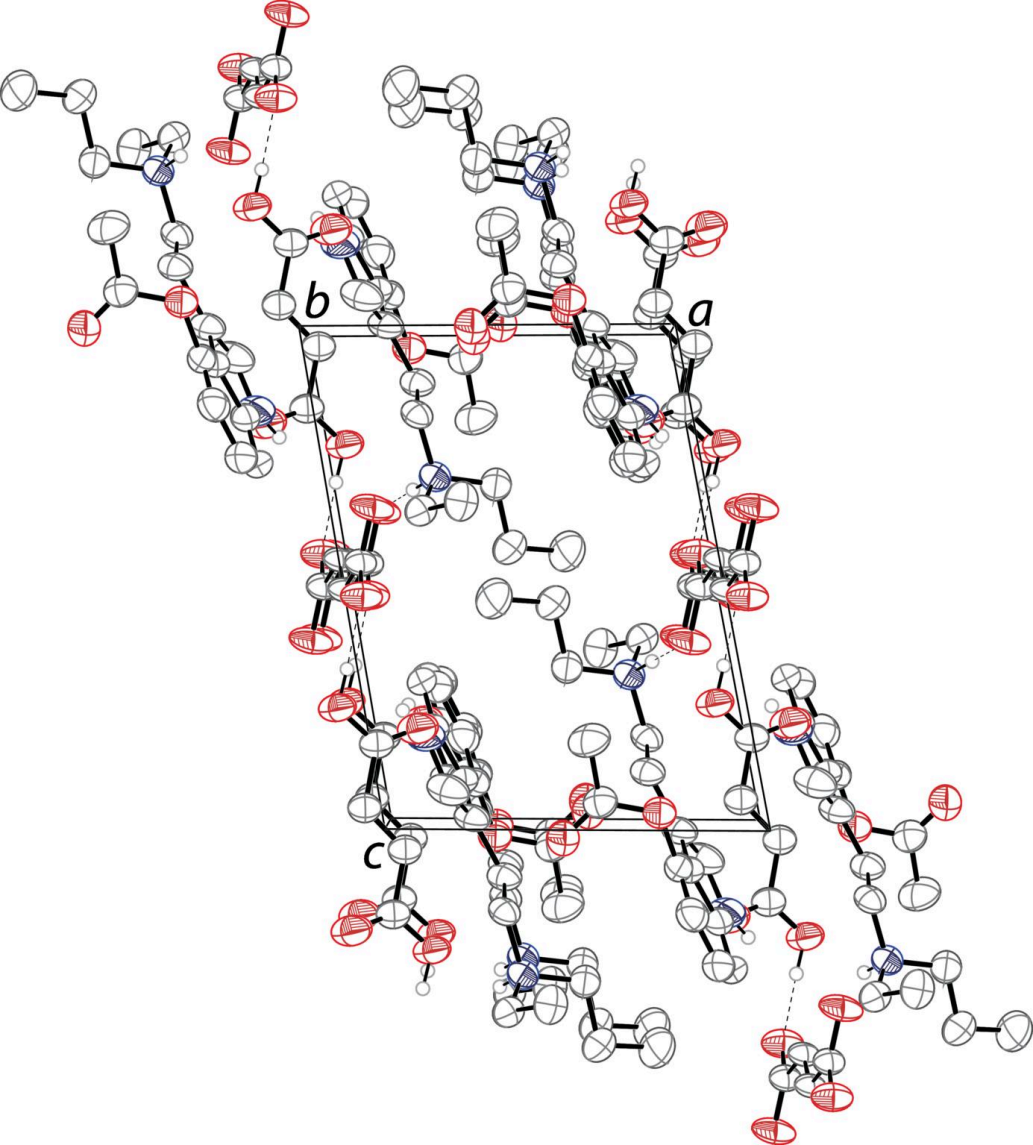
The crystal packing of the title compound viewed along the *b* axis. The hydrogen bonds are shown as dashed lines. Hydrogen atoms not involved in hydrogen bonds are omitted for clarity. Only one component of disorders are shown.

In the extended structure, the 4-acet­oxy-*N*-ethyl-*N*-*n*-propyl­tryptammonium cations, fumarate dianions and neutral fumaric acid mol­ecules are linked together in infinite one-dimensional chain propagating along [001]. The tryptammonium cations are linked to the fumarate dianions through N2—H2⋯O5 hydrogen bonds, and the fumaric acid mol­ecules are linked to the fumarate dianions through O4—H4*A*⋯O6 hydrogen bonds (Table 1 ▸). The packing of 4-AcO-EPT fumarate–fumaric acid is shown in Fig. 2 ▸.

In addition to the structure reported here, there have been ten other 4-acet­oxy­tryptamine structures reported in the literature, which include one mono­alkyl­tryptamine, 4-acet­oxy-*N*-methyl­tryptamine as its chloride salt (Glatfelter *et al.*, 2022*b* ▸), five di­alkyl­trypamines 4-acet­oxy-*N*,*N*-di­methyl­tryptamine as its hydro­fumarate (Chadeayne *et al.*, 2019*b* ▸) and fumarate (Chadeayne *et al.*, 2019*a* ▸) salts, 4-acet­oxy-*N*-methyl-*N*-ethyl­tryptamine and 4-acet­oxy-*N*-methyl-*N*-allyl­tryptamine as hydro­fumarate salts, and 4-acet­oxy-*N*,*N*-di­allyl­tryptamine as a fumarate-fumaric acid structure similar to that reported in this manuscript (Pham *et al.*, 2021 ▸). There are four tri­alkyl­tryptamine structures reported, 4-acet­oxy-*N*,*N*,*N*-tri­methyl­tryptamine (Chadeayne *et al.*, 2020 ▸), 4-acet­oxy-*N*,*N*-dimethyl-*N*-*n*-propyl­tryptamine, 4-acet­oxy-*N*,*N*-dimethyl-*N*-iso­propyl­tryptamine, 4-acet­oxy-*N*,*N*-dimethyl-*N*-ethyl­tryptamine all as their iodide salts (Glatfelter *et al.*, 2022*a* ▸). There are also three other 4-carb­oxy­lic ester prodrug structure reported, which are 4-propion­oxy-*N*,*N*-di­methyl­tryptamine as its hydro­fumarate salt (Glatfelter *et al.*, 2023 ▸) and two structures of the zwitterionic 4-glutarato-*N*,*N*-diiso­propyl­tryptamine (Naeem *et al.*, 2022 ▸).

## Synthesis and crystallization

Crystals of bis­(*N*-ethyl-*N*-*n*-propyl­tryptammonium) fumarate–fumaric acid were grown from the slow evaporation of an aqueous solution of ‘4-AcO-EPT fumarate’ obtained from ChemLogix.

## Refinement

Crystal data, data collection and structure refinement details are summarized in Table 2 ▸. Atoms H1, H2 and H4*A* were found from a Fourier difference map and allowed to refine with restrained N—H distances of 0.87 (1) Å and 1.20 *U*_eq_ of parent N atoms, and O—H distances of 0.88 (1) Å and 1.50 *U*_eq_ of parent O atoms. All other hydrogen atoms were placed in calculated positions with appropriate riding parameters. Ethyl and propyl groups showed overlap disorder with respect to each other and were treated using SADI C—C distance restrains, DELU rigid body restraints, and ISOR isotropic restraints.

## Supplementary Material

Crystal structure: contains datablock(s) I. DOI: 10.1107/S2414314623007794/hb4447sup1.cif

Structure factors: contains datablock(s) I. DOI: 10.1107/S2414314623007794/hb4447Isup2.hkl

CCDC reference: 2293403

Additional supporting information:  crystallographic information; 3D view; checkCIF report

## Figures and Tables

**Table 1:** Hydrogen-bond geometry (Å, °)

*D*—H⋯*A*	*D*—H	H⋯*A*	*D*⋯*A*	*D*—H⋯*A*
O4—H4*A*⋯O6	0.89 (1)	1.65 (1)	2.531 (2)	168 (3)
N2—H2⋯O5^i^	0.88 (1)	1.89 (1)	2.757 (2)	166 (2)

Symmetry code: (i) 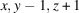 .

**Table 2:** Experimental details

Crystal data	
Chemical formula	C_17_H_25_N_2_O_2_^+^·0.5C_4_H_2_O_4_^2−^·0.5C_4_H_4_O_4_
*M*_r_	404.45
Crystal system, space group	Triclinic, *P* 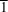
Temperature (K)	297
*a*, *b*, *c* (Å)	8.7642 (8), 10.8653 (9), 12.6564 (11)
α, β, γ (°)	65.094 (3), 75.354 (3), 76.718 (3)
*V* (Å^3^)	1047.12 (16)
*Z*	2
Radiation type	Mo *K*α
μ (mm^−1^)	0.09
Crystal size (mm)	0.22 × 0.2 × 0.12

Data collection	
Diffractometer	Bruker D8 Venture CMOS
Absorption correction	Multi-scan (*SADABS*; Krause *et al.*, 2015 ▸)
*T*_min_, *T*_max_	0.721, 0.745
No. of measured, independent and observed [*I* > 2σ(*I*)] reflections	28835, 4251, 3227
*R*_int_	0.041
(sin θ/λ)_max_ (Å^−1^)	0.626

Refinement	
*R*[*F* ^2^ > 2σ(*F* ^2^)], *wR*(*F* ^2^), *S*	0.056, 0.137, 1.03
No. of reflections	4251
No. of parameters	341
No. of restraints	115
H-atom treatment	H atoms treated by a mixture of independent and constrained refinement
Δρ_max_, Δρ_min_ (e Å^−3^)	0.35, −0.22

Computer programs: *APEX3* and *SAINT* (Bruker, 2018 ▸), *SHELXT2014/5* (Sheldrick, 2015*a* ▸), *SHELXL2019/3* (Sheldrick, 2015*b* ▸), *OLEX2* (Dolomanov *et al.*, 2009 ▸), and *publCIF* (Westrip, 2010 ▸).

## full crystallographic data

### Crystal data

**Table:**

C_17_H_25_N_2_O_2_^+^·0.5C_4_H_2_O_4_^2^^−^·0.5C_4_H_4_O_4_	*Z* = 2
*M**_r_* = 404.45	*F*(000) = 432
Triclinic, *P*1	*D*_x_ = 1.283 Mg m^−^^3^
*a* = 8.7642 (8) Å	Mo *K*α radiation, λ = 0.71073 Å
*b* = 10.8653 (9) Å	Cell parameters from 8239 reflections
*c* = 12.6564 (11) Å	θ = 2.8–26.3°
α = 65.094 (3)°	µ = 0.09 mm^−^^1^
β = 75.354 (3)°	*T* = 297 K
γ = 76.718 (3)°	BLOCK, colourless
*V* = 1047.12 (16) Å^3^	0.22 × 0.2 × 0.12 mm

### Data collection

**Table:**

Bruker D8 Venture CMOS diffractometer	3227 reflections with *I* > 2σ(*I*)
φ and ω scans	*R*_int_ = 0.041
Absorption correction: multi-scan (SADABS; Krause *et al.*, 2015)	θ_max_ = 26.4°, θ_min_ = 2.8°
*T*_min_ = 0.721, *T*_max_ = 0.745	*h* = −10→10
28835 measured reflections	*k* = −13→13
4251 independent reflections	*l* = −15→15

### Refinement

**Table:**

Refinement on *F*^2^	115 restraints
Least-squares matrix: full	Hydrogen site location: mixed
*R*[*F*^2^ > 2σ(*F*^2^)] = 0.056	H atoms treated by a mixture of independent and constrained refinement
*wR*(*F*^2^) = 0.137	*w* = 1/[σ^2^(*F*_o_^2^) + (0.0482*P*)^2^ + 0.5076*P*] where *P* = (*F*_o_^2^ + 2*F*_c_^2^)/3
*S* = 1.03	(Δ/σ)_max_ < 0.001
4251 reflections	Δρ_max_ = 0.35 e Å^−^^3^
341 parameters	Δρ_min_ = −0.22 e Å^−^^3^

### Special details

**Table:**

Geometry. All esds (except the esd in the dihedral angle between two l.s. planes) are estimated using the full covariance matrix. The cell esds are taken into account individually in the estimation of esds in distances, angles and torsion angles; correlations between esds in cell parameters are only used when they are defined by crystal symmetry. An approximate (isotropic) treatment of cell esds is used for estimating esds involving l.s. planes.

### Fractional atomic coordinates and isotropic or equivalent isotropic displacement parameters (Å^2^)

**Table:**

	*x*	*y*	*z*	*U*_iso_*/*U*_eq_	Occ. (<1)
O1	0.27647 (17)	−0.80720 (14)	1.03014 (13)	0.0591 (4)	
O2	0.5398 (2)	−0.8121 (2)	0.97964 (16)	0.0830 (6)	
O3	0.1315 (2)	−0.02323 (17)	0.80306 (14)	0.0773 (5)	
O4	−0.0658 (2)	0.14933 (15)	0.76151 (12)	0.0627 (4)	
H4A	−0.024 (3)	0.168 (3)	0.6856 (11)	0.094*	
O5	0.1155 (2)	0.21798 (14)	0.36799 (12)	0.0647 (4)	
O6	0.0480 (2)	0.23432 (14)	0.54237 (12)	0.0604 (4)	
N1	0.1435 (3)	−0.3427 (2)	0.82261 (18)	0.0772 (6)	
H1	0.096 (3)	−0.272 (2)	0.772 (2)	0.093*	
N2	0.2880 (2)	−0.56997 (17)	1.29957 (15)	0.0529 (4)	
H2	0.220 (2)	−0.6296 (18)	1.3266 (19)	0.063*	
C1	0.1785 (3)	−0.3645 (2)	0.9298 (2)	0.0744 (7)	
H1A	0.169952	−0.296392	0.958025	0.089*	
C2	0.1695 (2)	−0.4643 (2)	0.80871 (18)	0.0583 (6)	
C3	0.1561 (3)	−0.4964 (3)	0.71526 (19)	0.0694 (7)	
H3	0.123104	−0.428623	0.647012	0.083*	
C4	0.1927 (3)	−0.6296 (3)	0.7277 (2)	0.0700 (7)	
H4	0.187362	−0.652530	0.665853	0.084*	
C5	0.2379 (3)	−0.7321 (3)	0.8300 (2)	0.0629 (6)	
H5	0.259661	−0.822787	0.836792	0.075*	
C6	0.2508 (2)	−0.7010 (2)	0.92117 (17)	0.0508 (5)	
C7	0.2216 (2)	−0.5657 (2)	0.91297 (16)	0.0491 (5)	
C8	0.2275 (3)	−0.5003 (2)	0.98849 (17)	0.0558 (5)	
C9	0.2847 (3)	−0.5751 (2)	1.10637 (19)	0.0527 (8)	0.895 (7)
H9A	0.223769	−0.650132	1.155249	0.063*	0.895 (7)
H9B	0.395507	−0.614059	1.092468	0.063*	0.895 (7)
C10	0.2694 (4)	−0.4845 (3)	1.17281 (19)	0.0490 (8)	0.895 (7)
H10A	0.350332	−0.424109	1.135008	0.059*	0.895 (7)
H10B	0.165866	−0.428210	1.170602	0.059*	0.895 (7)
C9A	0.182 (3)	−0.524 (3)	1.1193 (10)	0.069 (8)	0.105 (7)
H9AA	0.093424	−0.456113	1.130631	0.083*	0.105 (7)
H9AB	0.149277	−0.613887	1.164253	0.083*	0.105 (7)
C10A	0.320 (2)	−0.514 (3)	1.1640 (9)	0.089 (12)	0.105 (7)
H10C	0.337116	−0.419607	1.132379	0.107*	0.105 (7)
H10D	0.415098	−0.566634	1.136772	0.107*	0.105 (7)
C11	0.4486 (5)	−0.6529 (6)	1.3152 (4)	0.0547 (11)	0.741 (6)
H11A	0.528096	−0.591640	1.285811	0.066*	0.741 (6)
H11B	0.474027	−0.708899	1.268550	0.066*	0.741 (6)
C12	0.4558 (6)	−0.7450 (5)	1.4437 (4)	0.0717 (12)	0.741 (6)
H12A	0.365280	−0.795179	1.477680	0.086*	0.741 (6)
H12B	0.450747	−0.689565	1.488122	0.086*	0.741 (6)
C13	0.6093 (5)	−0.8451 (4)	1.4525 (4)	0.1003 (16)	0.741 (6)
H13A	0.605799	−0.911371	1.532280	0.150*	0.741 (6)
H13B	0.697996	−0.796160	1.430637	0.150*	0.741 (6)
H13C	0.620986	−0.891082	1.400040	0.150*	0.741 (6)
C14	0.2341 (7)	−0.4813 (5)	1.3701 (4)	0.0505 (10)	0.741 (6)
H14A	0.215332	−0.540227	1.453431	0.061*	0.741 (6)
H14B	0.133039	−0.426905	1.350644	0.061*	0.741 (6)
C15	0.3488 (6)	−0.3845 (5)	1.3515 (5)	0.0649 (12)	0.741 (6)
H15A	0.300869	−0.327687	1.395530	0.097*	0.741 (6)
H15B	0.372168	−0.327944	1.268845	0.097*	0.741 (6)
H15C	0.445657	−0.437161	1.378303	0.097*	0.741 (6)
C11A	0.266 (3)	−0.493 (2)	1.3766 (17)	0.090 (7)	0.259 (6)
H11C	0.282534	−0.553750	1.456143	0.108*	0.259 (6)
H11D	0.160829	−0.439951	1.380442	0.108*	0.259 (6)
C12A	0.394 (2)	−0.3992 (17)	1.3153 (14)	0.077 (4)	0.259 (6)
H12C	0.499877	−0.450786	1.312186	0.093*	0.259 (6)
H12D	0.378019	−0.336885	1.235901	0.093*	0.259 (6)
C13A	0.3632 (14)	−0.3249 (12)	1.3981 (10)	0.084 (3)	0.259 (6)
H13D	0.442001	−0.265190	1.373427	0.125*	0.259 (6)
H13E	0.368967	−0.390507	1.477188	0.125*	0.259 (6)
H13F	0.259198	−0.271806	1.396039	0.125*	0.259 (6)
C14A	0.439 (2)	−0.671 (2)	1.3038 (19)	0.100 (9)	0.259 (6)
H14C	0.526145	−0.616857	1.265809	0.121*	0.259 (6)
H14D	0.437114	−0.716748	1.253028	0.121*	0.259 (6)
C15A	0.487 (3)	−0.783 (2)	1.4176 (17)	0.129 (8)	0.259 (6)
H15D	0.480279	−0.871008	1.419487	0.194*	0.259 (6)
H15E	0.416676	−0.769476	1.484596	0.194*	0.259 (6)
H15F	0.594408	−0.779507	1.420176	0.194*	0.259 (6)
C16	0.4261 (3)	−0.8539 (2)	1.0513 (2)	0.0568 (5)	
C17	0.4260 (4)	−0.9596 (2)	1.1741 (2)	0.0785 (7)	
H17A	0.346455	−0.928264	1.228438	0.118*	
H17B	0.528807	−0.974856	1.194762	0.118*	
H17C	0.402813	−1.043724	1.177925	0.118*	
C18	0.0174 (3)	0.0486 (2)	0.83333 (17)	0.0506 (5)	
C19	−0.0428 (3)	0.0340 (2)	0.95887 (17)	0.0524 (5)	
H19	−0.145138	0.075199	0.978794	0.063*	
C20	0.0593 (2)	0.17179 (18)	0.47610 (16)	0.0453 (4)	
C21	0.0000 (2)	0.03642 (17)	0.52990 (15)	0.0434 (4)	
H21	−0.039388	0.001939	0.610781	0.052*	

### Atomic displacement parameters (Å^2^)

**Table:**

	*U*^11^	*U*^22^	*U*^33^	*U*^12^	*U*^13^	*U*^23^
O1	0.0588 (9)	0.0495 (8)	0.0537 (8)	−0.0130 (7)	−0.0083 (7)	−0.0035 (6)
O2	0.0573 (10)	0.0979 (13)	0.0694 (11)	−0.0046 (9)	−0.0113 (8)	−0.0121 (10)
O3	0.0949 (13)	0.0707 (10)	0.0511 (9)	0.0132 (9)	−0.0160 (9)	−0.0199 (8)
O4	0.0790 (11)	0.0586 (9)	0.0415 (8)	−0.0005 (8)	−0.0137 (7)	−0.0136 (7)
O5	0.0983 (12)	0.0531 (8)	0.0384 (7)	−0.0426 (8)	0.0083 (7)	−0.0087 (6)
O6	0.0938 (11)	0.0453 (8)	0.0433 (8)	−0.0271 (7)	−0.0052 (7)	−0.0133 (6)
N1	0.0837 (15)	0.0567 (12)	0.0508 (12)	0.0138 (10)	−0.0049 (10)	0.0016 (9)
N2	0.0529 (10)	0.0473 (9)	0.0487 (9)	−0.0206 (7)	−0.0061 (8)	−0.0041 (7)
C1	0.0951 (19)	0.0511 (12)	0.0548 (14)	−0.0016 (12)	0.0072 (12)	−0.0151 (10)
C2	0.0460 (11)	0.0620 (13)	0.0434 (11)	0.0019 (9)	−0.0040 (9)	−0.0049 (9)
C3	0.0475 (12)	0.102 (2)	0.0396 (11)	−0.0074 (12)	−0.0119 (9)	−0.0088 (12)
C4	0.0571 (14)	0.100 (2)	0.0534 (13)	−0.0204 (13)	−0.0075 (11)	−0.0270 (13)
C5	0.0560 (13)	0.0737 (14)	0.0624 (14)	−0.0216 (11)	−0.0037 (10)	−0.0270 (12)
C6	0.0431 (10)	0.0546 (11)	0.0450 (11)	−0.0123 (8)	−0.0076 (8)	−0.0072 (9)
C7	0.0442 (10)	0.0512 (11)	0.0377 (10)	−0.0062 (8)	−0.0053 (8)	−0.0051 (8)
C8	0.0665 (13)	0.0468 (11)	0.0402 (10)	−0.0101 (9)	0.0004 (9)	−0.0079 (8)
C9	0.0639 (17)	0.0400 (12)	0.0419 (12)	−0.0031 (10)	−0.0032 (10)	−0.0096 (9)
C10	0.0513 (16)	0.0403 (12)	0.0436 (13)	−0.0108 (11)	0.0010 (10)	−0.0080 (10)
C9A	0.068 (11)	0.075 (11)	0.070 (11)	−0.018 (8)	−0.013 (8)	−0.028 (8)
C10A	0.070 (13)	0.094 (15)	0.092 (15)	−0.033 (9)	−0.031 (9)	−0.004 (9)
C11	0.0516 (19)	0.0473 (18)	0.058 (2)	−0.0096 (14)	−0.0113 (15)	−0.0116 (17)
C12	0.066 (2)	0.073 (2)	0.063 (2)	0.0052 (19)	−0.0225 (18)	−0.0152 (19)
C13	0.077 (3)	0.088 (3)	0.094 (3)	0.008 (2)	−0.025 (2)	0.001 (2)
C14	0.057 (2)	0.0467 (17)	0.0506 (19)	−0.0126 (15)	−0.0136 (16)	−0.0163 (14)
C15	0.064 (3)	0.057 (2)	0.085 (3)	−0.0126 (18)	−0.019 (2)	−0.033 (2)
C11A	0.082 (9)	0.097 (9)	0.095 (9)	−0.022 (5)	−0.027 (5)	−0.028 (5)
C12A	0.081 (7)	0.071 (6)	0.079 (6)	−0.020 (4)	−0.015 (5)	−0.024 (4)
C13A	0.089 (7)	0.081 (6)	0.082 (7)	−0.016 (5)	−0.014 (5)	−0.032 (5)
C14A	0.115 (10)	0.089 (9)	0.106 (9)	−0.035 (5)	−0.011 (5)	−0.040 (5)
C15A	0.134 (12)	0.127 (11)	0.113 (10)	0.023 (9)	−0.024 (8)	−0.053 (7)
C16	0.0644 (14)	0.0473 (11)	0.0556 (12)	−0.0028 (10)	−0.0133 (11)	−0.0178 (9)
C17	0.101 (2)	0.0540 (13)	0.0629 (15)	0.0052 (13)	−0.0234 (14)	−0.0089 (11)
C18	0.0631 (13)	0.0458 (10)	0.0445 (11)	−0.0113 (9)	−0.0088 (9)	−0.0173 (9)
C19	0.0596 (12)	0.0467 (11)	0.0471 (11)	−0.0105 (9)	−0.0083 (9)	−0.0135 (8)
C20	0.0537 (11)	0.0373 (9)	0.0385 (10)	−0.0149 (8)	−0.0042 (8)	−0.0064 (8)
C21	0.0521 (10)	0.0389 (9)	0.0312 (9)	−0.0166 (8)	−0.0007 (8)	−0.0042 (7)

### Geometric parameters (Å, º)

**Table:**

O1—C6	1.408 (2)	C10A—H10D	0.9700
O1—C16	1.343 (3)	C11—H11A	0.9700
O2—C16	1.194 (3)	C11—H11B	0.9700
O3—C18	1.206 (3)	C11—C12	1.518 (5)
O4—H4A	0.889 (10)	C12—H12A	0.9700
O4—C18	1.299 (2)	C12—H12B	0.9700
O5—C20	1.250 (2)	C12—C13	1.518 (5)
O6—C20	1.258 (2)	C13—H13A	0.9600
N1—H1	0.869 (10)	C13—H13B	0.9600
N1—C1	1.377 (3)	C13—H13C	0.9600
N1—C2	1.366 (3)	C14—H14A	0.9700
N2—H2	0.880 (10)	C14—H14B	0.9700
N2—C10	1.507 (3)	C14—C15	1.526 (5)
N2—C10A	1.533 (10)	C15—H15A	0.9600
N2—C11	1.497 (3)	C15—H15B	0.9600
N2—C14	1.501 (3)	C15—H15C	0.9600
N2—C11A	1.490 (8)	C11A—H11C	0.9700
N2—C14A	1.510 (8)	C11A—H11D	0.9700
C1—H1A	0.9300	C11A—C12A	1.516 (9)
C1—C8	1.364 (3)	C12A—H12C	0.9700
C2—C3	1.404 (3)	C12A—H12D	0.9700
C2—C7	1.419 (3)	C12A—C13A	1.515 (9)
C3—H3	0.9300	C13A—H13D	0.9600
C3—C4	1.357 (4)	C13A—H13E	0.9600
C4—H4	0.9300	C13A—H13F	0.9600
C4—C5	1.384 (3)	C14A—H14C	0.9700
C5—H5	0.9300	C14A—H14D	0.9700
C5—C6	1.368 (3)	C14A—C15A	1.525 (11)
C6—C7	1.396 (3)	C15A—H15D	0.9600
C7—C8	1.426 (3)	C15A—H15E	0.9600
C8—C9	1.519 (3)	C15A—H15F	0.9600
C8—C9A	1.524 (10)	C16—C17	1.494 (3)
C9—H9A	0.9700	C17—H17A	0.9600
C9—H9B	0.9700	C17—H17B	0.9600
C9—C10	1.508 (3)	C17—H17C	0.9600
C10—H10A	0.9700	C18—C19	1.494 (3)
C10—H10B	0.9700	C19—C19^i^	1.298 (4)
C9A—H9AA	0.9700	C19—H19	0.9300
C9A—H9AB	0.9700	C20—C21	1.492 (2)
C9A—C10A	1.491 (10)	C21—C21^ii^	1.305 (4)
C10A—H10C	0.9700	C21—H21	0.9300
			
C16—O1—C6	119.21 (16)	C11—C12—H12B	109.6
C18—O4—H4A	113.2 (19)	C11—C12—C13	110.1 (4)
C1—N1—H1	131.7 (19)	H12A—C12—H12B	108.2
C2—N1—H1	117.7 (19)	C13—C12—H12A	109.6
C2—N1—C1	109.69 (18)	C13—C12—H12B	109.6
C10—N2—H2	105.7 (15)	C12—C13—H13A	109.5
C10A—N2—H2	108.6 (18)	C12—C13—H13B	109.5
C11—N2—H2	105.9 (15)	C12—C13—H13C	109.5
C11—N2—C10	114.7 (3)	H13A—C13—H13B	109.5
C11—N2—C14	114.0 (4)	H13A—C13—H13C	109.5
C14—N2—H2	106.6 (15)	H13B—C13—H13C	109.5
C14—N2—C10	109.2 (3)	N2—C14—H14A	108.4
C11A—N2—H2	112.0 (16)	N2—C14—H14B	108.4
C11A—N2—C10A	127.9 (15)	N2—C14—C15	115.6 (3)
C11A—N2—C14A	114.3 (14)	H14A—C14—H14B	107.4
C14A—N2—H2	97.4 (18)	C15—C14—H14A	108.4
C14A—N2—C10A	90.9 (7)	C15—C14—H14B	108.4
N1—C1—H1A	124.9	C14—C15—H15A	109.5
C8—C1—N1	110.2 (2)	C14—C15—H15B	109.5
C8—C1—H1A	124.9	C14—C15—H15C	109.5
N1—C2—C3	131.9 (2)	H15A—C15—H15B	109.5
N1—C2—C7	106.0 (2)	H15A—C15—H15C	109.5
C3—C2—C7	122.1 (2)	H15B—C15—H15C	109.5
C2—C3—H3	121.0	N2—C11A—H11C	111.2
C4—C3—C2	117.9 (2)	N2—C11A—H11D	111.2
C4—C3—H3	121.0	N2—C11A—C12A	103.0 (10)
C3—C4—H4	119.2	H11C—C11A—H11D	109.1
C3—C4—C5	121.7 (2)	C12A—C11A—H11C	111.2
C5—C4—H4	119.2	C12A—C11A—H11D	111.2
C4—C5—H5	119.8	C11A—C12A—H12C	111.9
C6—C5—C4	120.4 (2)	C11A—C12A—H12D	111.9
C6—C5—H5	119.8	H12C—C12A—H12D	109.6
C5—C6—O1	119.70 (19)	C13A—C12A—C11A	99.6 (9)
C5—C6—C7	121.20 (19)	C13A—C12A—H12C	111.9
C7—C6—O1	118.72 (18)	C13A—C12A—H12D	111.9
C2—C7—C8	108.50 (19)	C12A—C13A—H13D	109.5
C6—C7—C2	116.6 (2)	C12A—C13A—H13E	109.5
C6—C7—C8	134.88 (18)	C12A—C13A—H13F	109.5
C1—C8—C7	105.5 (2)	H13D—C13A—H13E	109.5
C1—C8—C9	130.6 (2)	H13D—C13A—H13F	109.5
C1—C8—C9A	105.2 (12)	H13E—C13A—H13F	109.5
C7—C8—C9	123.84 (18)	N2—C14A—H14C	106.3
C7—C8—C9A	138.6 (9)	N2—C14A—H14D	106.3
C8—C9—H9A	108.9	N2—C14A—C15A	124.1 (16)
C8—C9—H9B	108.9	H14C—C14A—H14D	106.4
H9A—C9—H9B	107.7	C15A—C14A—H14C	106.3
C10—C9—C8	113.5 (2)	C15A—C14A—H14D	106.3
C10—C9—H9A	108.9	C14A—C15A—H15D	109.5
C10—C9—H9B	108.9	C14A—C15A—H15E	109.5
N2—C10—C9	110.48 (19)	C14A—C15A—H15F	109.5
N2—C10—H10A	109.6	H15D—C15A—H15E	109.5
N2—C10—H10B	109.6	H15D—C15A—H15F	109.5
C9—C10—H10A	109.6	H15E—C15A—H15F	109.5
C9—C10—H10B	109.6	O1—C16—C17	110.6 (2)
H10A—C10—H10B	108.1	O2—C16—O1	122.7 (2)
C8—C9A—H9AA	109.5	O2—C16—C17	126.7 (2)
C8—C9A—H9AB	109.5	C16—C17—H17A	109.5
H9AA—C9A—H9AB	108.0	C16—C17—H17B	109.5
C10A—C9A—C8	110.9 (12)	C16—C17—H17C	109.5
C10A—C9A—H9AA	109.5	H17A—C17—H17B	109.5
C10A—C9A—H9AB	109.5	H17A—C17—H17C	109.5
N2—C10A—H10C	109.5	H17B—C17—H17C	109.5
N2—C10A—H10D	109.5	O3—C18—O4	124.62 (19)
C9A—C10A—N2	110.9 (12)	O3—C18—C19	123.65 (19)
C9A—C10A—H10C	109.5	O4—C18—C19	111.71 (18)
C9A—C10A—H10D	109.5	C18—C19—H19	118.8
H10C—C10A—H10D	108.1	C19^i^—C19—C18	122.3 (3)
N2—C11—H11A	109.2	C19^i^—C19—H19	118.8
N2—C11—H11B	109.2	O5—C20—O6	123.40 (16)
N2—C11—C12	112.2 (3)	O5—C20—C21	118.75 (17)
H11A—C11—H11B	107.9	O6—C20—C21	117.85 (16)
C12—C11—H11A	109.2	C20—C21—H21	118.1
C12—C11—H11B	109.2	C21^ii^—C21—C20	123.8 (2)
C11—C12—H12A	109.6	C21^ii^—C21—H21	118.1
			
O1—C6—C7—C2	−169.44 (17)	C4—C5—C6—C7	−1.1 (3)
O1—C6—C7—C8	8.5 (3)	C5—C6—C7—C2	3.5 (3)
O3—C18—C19—C19^i^	16.7 (4)	C5—C6—C7—C8	−178.5 (2)
O4—C18—C19—C19^i^	−162.0 (2)	C6—O1—C16—O2	−2.6 (3)
O5—C20—C21—C21^ii^	−1.5 (4)	C6—O1—C16—C17	176.42 (19)
O6—C20—C21—C21^ii^	177.7 (2)	C6—C7—C8—C1	−177.5 (2)
N1—C1—C8—C7	−0.2 (3)	C6—C7—C8—C9	4.6 (4)
N1—C1—C8—C9	177.5 (2)	C6—C7—C8—C9A	−40.9 (16)
N1—C1—C8—C9A	−152.1 (9)	C7—C2—C3—C4	0.9 (3)
N1—C2—C3—C4	179.3 (2)	C7—C8—C9—C10	−176.3 (2)
N1—C2—C7—C6	177.81 (18)	C7—C8—C9A—C10A	128.1 (16)
N1—C2—C7—C8	−0.7 (2)	C8—C9—C10—N2	165.70 (19)
N2—C11—C12—C13	169.6 (5)	C8—C9A—C10A—N2	−167.0 (16)
N2—C11A—C12A—C13A	179.8 (14)	C10—N2—C11—C12	−175.5 (4)
C1—N1—C2—C3	−178.1 (2)	C10—N2—C14—C15	−75.4 (5)
C1—N1—C2—C7	0.6 (3)	C10A—N2—C11A—C12A	−42 (2)
C1—C8—C9—C10	6.4 (4)	C10A—N2—C14A—C15A	−173 (3)
C1—C8—C9A—C10A	−95 (2)	C11—N2—C10—C9	64.1 (4)
C2—N1—C1—C8	−0.2 (3)	C11—N2—C14—C15	54.3 (6)
C2—C3—C4—C5	1.7 (3)	C14—N2—C10—C9	−166.6 (3)
C2—C7—C8—C1	0.5 (2)	C14—N2—C11—C12	57.5 (6)
C2—C7—C8—C9	−177.3 (2)	C11A—N2—C10A—C9A	−114 (3)
C2—C7—C8—C9A	137.2 (16)	C11A—N2—C14A—C15A	54 (3)
C3—C2—C7—C6	−3.4 (3)	C14A—N2—C10A—C9A	124 (3)
C3—C2—C7—C8	178.10 (19)	C14A—N2—C11A—C12A	70 (2)
C3—C4—C5—C6	−1.7 (3)	C16—O1—C6—C5	94.6 (2)
C4—C5—C6—O1	171.77 (19)	C16—O1—C6—C7	−92.4 (2)

Symmetry codes: (i) −*x*, −*y*, −*z*+2; (ii) −*x*, −*y*, −*z*+1.

### Hydrogen-bond geometry (Å, º)

**Table:**

*D*—H···*A*	*D*—H	H···*A*	*D*···*A*	*D*—H···*A*
O4—H4*A*···O6	0.89 (1)	1.65 (1)	2.531 (2)	168 (3)
N2—H2···O5^iii^	0.88 (1)	1.89 (1)	2.757 (2)	166 (2)
C9—H9*A*···O5^iii^	0.97	2.51	3.316 (3)	141
C10—H10*A*···O2^iv^	0.97	2.63	3.537 (3)	156
C9*A*—H9*AB*···O5^iii^	0.97	2.44	3.24 (2)	139
C9*A*—H9*AB*···N1^v^	0.97	2.67	3.28 (2)	121
C12*A*—H12*D*···O2^iv^	0.97	2.53	3.441 (17)	156

Symmetry codes: (iii) *x*, *y*−1, *z*+1; (iv) −*x*+1, −*y*−1, −*z*+2; (v) −*x*, −*y*−1, −*z*+2.
